# The lncRNA HCG4 regulates the RIG-I-mediated IFN production to suppress H1N1 swine influenza virus replication

**DOI:** 10.3389/fmicb.2023.1324218

**Published:** 2024-01-11

**Authors:** Jinghua Cheng, Jie Tao, Benqiang Li, Ying Shi, Huili Liu

**Affiliations:** ^1^Institute of Animal Science and Veterinary Medicine, Shanghai Academy of Agricultural Sciences, Shanghai, China; ^2^Shanghai Key Laboratory of Agricultural Genetic Breeding, Shanghai, China; ^3^Shanghai Engineering Research Center of Pig Breeding, Shanghai, China

**Keywords:** swine influenza virus, NS1 protein, lncRNA, IFN, RIG-I ubiquitination

## Abstract

Influenza A virus (IAV) non-structural protein 1 (NS1) is a virulence factor that allows the virus to replicate efficiently by suppressing host innate immune responses. Previously, we demonstrated that the serine (S) at position 42 of NS1 in H1N1 swine influenza virus (SIV) is a critical residue in interferon (IFN) resistance, thus facilitating viral infections. Here, by lncRNA-seq, a total of 153 differentially expressed lncRNAs were identified, and the lncRNA HCG4 was selected due to its significantly higher expression after infection with the NS1 S42P mutant virus. Overexpression of HCG4 enhanced IFN-β production and suppressed SIV infection, highlighting the potential antiviral activity of HCG4 against SIV. Further investigation suggested that HCG4 served as a positive feedback mediator for RIG-I signaling. It alleviated the inhibitory effect on RIG-I K63-linked ubiquitination by NS1 protein, thereby resulting in an increase in RIG-I-mediated IFN production. Taken together, our findings demonstrate that HCG4 modulates the innate immune response to SIV infection through K63-linked RIG-I ubiquitination, providing insights into the role of lncRNAs in controlling viral infections.

## 1 Introduction

Influenza A virus (IAV) is a member of the Orthomyxoviridae family of RNA viruses that can infect numerous animal species, including humans, pigs, and domestic fowl. Encoded by gene segment 8, non-structural protein 1 (NS1) acts as a virulence factor by inhibiting host immune responses (Webster et al., 1992; Jackson et al., 2008). Several key amino acids involved in NS1 mutations or deletions (e.g., S42P, S42G, D92E, D92Y, I106M, and A149V) cause defects in viral fitness, including replication suppressed and incapable of exert antiviral response (Seo et al., 2002; Li et al., 2006; Jiao et al., 2008; Ayllon et al., 2014). Among these, the effects of amino acid residues at position 42 have been reported in many subtypes of influenza virus. In our previous study, we constructed different NS1 mutant viruses of the A/swine/Shanghai/3/2014 (H1N1) strain and found that the S42 residue is the key amino acid in regulating the host IFN response, thus facilitating virus replication (Cheng et al., 2018). However, the mechanism by which the NS1 protein of IAV exerts its IFN antagonistic properties is not yet fully understood.

Studies on virus-related host immune responses over the past several decades have focused mainly on genes or proteins. In recent years, a wide variety of host long non-coding RNAs (lncRNAs) have been confirmed to be a novel group of regulatory molecules that also participate in immune response-related biological processes.(Moran et al., 2012; Dey et al., 2014). LncRNAs are a class of non-coding RNAs with a length of over 200 nucleotides (nt) (Ernst and Morton, 2013). Usually, they function by enhancing or inhibiting the expression of neighboring protein-coding genes. They are in the cytoplasm or nucleus and play an important role in biological activities and disease development by regulating target genes, thereby affecting their gene expression (Ouyang et al., 2014; Meng et al., 2017). Notably, that viral infections may lead to the differential expression of host lncRNAs, and some differentially expressed host lncRNAs have been identified to exert antiviral actions involved in different immune signaling pathways (Fortes and Morris, 2016; Qian et al., 2016; Ma et al., 2017). They modulate diverse and multilayered immune checkpoints through activation or repression of innate immune signaling components (Fitzgerald and Caffrey, 2014; Heward and Lindsay, 2014).

The IFN-mediated innate immune response constitutes the initial defense against viral invasions. Emerging evidence points to the involvement of lncRNAs as key regulators of the IFN response related to innate immunity. For example, Kambara et al. (2014) reported a negative regulator of the IFN response, named lncRNA CMPK2, which can be induced by IFN in diverse cell types. Knockdown of lncRNA-CMPK2 can affect hepatitis C virus (HCV) replication through JAK-STAT signaling activation, which upregulated the transcription of several antiviral IFN-stimulated genes (ISGs) (Kambara et al., 2014). Likewise, Liu et al. (2020) identified an abundant nuclear lncRNA, Malat1, in macrophages to be a negative regulator of antiviral type I IFN production, thereby maintaining immune homeostasis. Conversely, lncRNA nuclear paraspeckle assembly transcript 1 (NEAT1) was reported to display positive feedback for IFN production in host cells during hantavirus (HTNV) infection. It was markedly upregulated by HTNV infection, and promoted IFN signaling, thus enhancing the host anti-hantaviral innate immune responses (Ma et al., 2017). Immunologically, upon virus infection, the host pathogen recognition receptors (PRRs), including Toll-like receptors (TLRs) and RIG-I like receptors (RLRs), detect the viral nucleic acids and trigger a series of signaling cascades that lead to the production and secretion of type I IFN. The negative-sense RNA genome of IAV is strictly recognized by RLRs, once activated, its N-terminal CARDs are exposed to recruit the mitochondrial localized virus-induced signaling adaptor MAVS, thereby resulting in the activation of IFN-regulated factors and the induction of IFNs (Iwasaki and Pillai, 2014). The pivotal role of the RIG-I-mediated antiviral response in IAV has been studied for a long time, but the functions and underlying mechanisms of lncRNAs in RIG-I signaling mediated antiviral processes have only recently been studied and are scarcely reported.

Here, we extended those earlier studies by utilizing an RNA sequencing approach that enables us to capture the different expression profiles of lncRNAs in A549 cells upon infection with H1N1 SIV and its NS1 mutant. We found that the lncRNA HCG4 was markedly upregulated by NS1 mutant infection. Silencing HCG4 *in vitro* attenuated RIG-I-triggered IFN-β expression and promoted SIV replication whereas HCG4 overexpression *in vitro* enhanced IFN-β production and inhibited SIV replication. Furthermore, we revealed that HCG4 alleviated the inhibitory effect on RIG-I K63-linked ubiquitination by the NS1 protein, thereby resulting in an increase in IFN-β production and supporting a more potent innate immune response against SIV infection. This study focused on the lncRNAs involved in host–virus interactions and revealed the underlying regulatory mechanisms of host lncRNAs in the innate immune response.

## 2 Materials and methods

### 2.1 Cells, viruses, and antibodies

The A/swine/Shanghai/3/2014 (H1N1) strain was isolated in our laboratory from pigs with clinical symptoms of swine influenza. Recombinant SH/2014 (rSH/2014) and rSH/2014 NS1 S42P (NS1 mutant of SH/2014) were constructed and rescued as described in a previous study (Cheng et al., 2018). A549 cells, MDCK cells and 293T cells were obtained from ATCC (Manassas, VA, USA) and maintained in DMEM (Gibco, Grand Island, NY, USA) supplemented with 10% fetal bovine serum (FBS) (Invitrogen, Carlsbad, CA, USA) at 37°C with 5% CO_2_. Antibodies against RIG-I, p-IRF3, p-STAT1, p-STAT2, K63-linked ubiquitin and β-actin were purchased from Cell Signaling Technology (Danvers, MA, USA). Anti-HA and anti-Flag antibodies were purchased from Sigma-Aldrich (St Louis, MO, USA). Antibodies against IAV NS1 and NP protein were purchased from Santa Cruz Biotechnology (Dallas, TX, USA).

### 2.2 High-throughput sequencing and validation of different expressed lncRNAs by qRT-PCR

After infection with rSH/2014 or rSH/2014 NS1 S42P for 24 h, total RNA was extracted from A549 cells using TRIzol (Invitrogen) according to the manufacturer’s instructions. The quality of the RNA samples was determined using an Agilent Bioanalyzer (Agilent Technologies, Santa Clara, CA, USA). High quality RNA was selected for library construction with the TruSeq RNA sample preparation kit (Illumina, San Diego, CA, USA) and sequencing was performed on an Illumina HiSeq 2000 instrument. RNA sequencing (RNA-seq) reads were aligned to the human genome (hg19) by using Hisat2. HTseq-count was used to count the reads mapped to each gene. Differentially expressed genes were analyzed by using DESeq2. DEGs were selected based on an adjusted *p*-value of 0.05 and at least a twofold difference in expression levels. The bioinformatics analysis was supported by RiboBio Biotechnology Co., Ltd. (Guangzhou, China).

To verify the accuracy of RNA-Seq, quantitative real-time PCR (qRT–PCR) was used to investigate the relative levels of differentially expressed lncRNAs. Total RNA was extracted from rSH/2014-infected, mutant-infected and uninfected cells at 24 h.p.i. using TRIzol reagent. cDNA synthesis was performed using the HiScript II 1st Strand cDNA Synthesis Kit (Vazyme Biotech, Nanjing, China) according to the manufacturer’s protocol. The sequences of the RNA primers are shown in Table 1. qRT–PCR was performed using SYBR Premix Ex Taq II (TaKaRa Bio, Shiga, Japan) on a 7500 Real Time PCR System apparatus. The PCR cycles were as follows: 95°C for 5 min followed by 40 cycles of 95°C for 15 s and 60°C for 30 s. Reactions were performed in triplicate. The relative expression was calculated by the 2^–ΔΔCt^ method (Schmittgen and Livak, 2008).

**TABLE 1:** Primers used for qRT–PCR.

Gene name	Forward primer	Reverse primer
THAP7-AS1	GGCCAACTGCAAA GAACACT	GAGGGAAGGAATT GGAAAGC
MAPT-IT1	CCCAATGGAGATGG CTCTAA	AGCCTACAGCAAGC CTTCAG
LOC105374789	ACGATGCTTGGAA CGTGATT	TGCTGGACTTCTG AAACAACA
HCG4	GATGTGGAGGGGTAG TGACC	CAAGGCCAAAGCAC AGTTTT
LOC105375363	TTCAAAGCCTCCAC GACTCT	CTGAGACTCAAGG CCGTCTC
USP2-AS1	TTTTGAACCTGGGA ACCTTG	ATTCCCACAAATTC CCATCA
SSTR5-AS1	AGGATTCAGGCTCA GGGAAT	CCACCCAAATCTTG CTGAAT
LOC112268182	CAGTGTGGCTCTG GATAGCA	CTAATGCCTGT GTGCCAATG
NP	TGTGTATGGACCTGC CGTAGC	CCATCCACACCAGTTGA CTCTTG
IFN-β	GCTGGAATGAG ACTATT GTTGAGA	CAGTTTCGGAGGTAACC TGTAAG
β-actin	GCACCGTCAAGGC TGAGAAC	TGGTGAAGACGC CAGTGGA

### 2.3 Plasmids and siRNA

The cDNA encoding full-length HCG4 was synthesized (RiboBio) and subcloned into the *Xho*I and *Eco*RI sites of the pcDNA3.1 vector (Invitrogen). Myc-RIG-I and HA-Ub-K63 were purchased from MiaoLing Biology (Wuhan, China). For silencing of HCG4 expression, HCG4-specific siRNAs.

(siHCG4:5′-GAAGCUAUGUUUGGAAUUATT-3′; non-targeting control siRNA: 5′-UUCUCCGAACGUGUCACGUTT-3′) were purchased from Gene Pharma Co. (Shanghai, China). Transfection plasmids or siRNAs at the indicated concentrations were transfected into A549 cells using Lipo3000 reagent (Thermo Fisher Scientific, Waltham, MA, USA).

### 2.4 TCID_50_ assay

MDCK cells were plated in 96 well plates and grown overnight. The virus was serially 10-fold diluted and inoculated onto MDCK cell monolayers. After incubation at 37°C for 1 h, the virus suspensions were removed and replaced with serum-free DMEM containing 2 μg/ml TPCK trypsin (Promega, Madison, WI, USA). At 12, 24, 36, 48, 60, and 72 h.p.i., the cytopathic effect (CPE) was observed microscopically and the TCID_50_ value was calculated using the Reed-Muench method (LaBarre and Lowy, 2001).

### 2.5 Fluorescence *in situ* hybridization (FISH) and immunofluorescence assays (IFA)

Fluorescence *in situ* hybridization was performed to determine the distribution of HCG4 in cells with a FISH kit (RiboBio) according to the manufacturer’s instructions. Briefly, cells seeded on coverslips were fixed in 4% paraformaldehyde for 30 min, and permeabilized with 0.5% Triton X-100 for 15 min at room temperature. Then, the cells were hybridized with 40 nM HCG4 probe in hybridization buffer overnight at 37°C. The probes for U6 and 18S mRNA were used as controls. The coverslips were then washed with 4 × , 2 × , and 1 × saline sodium citrate (SSC), and nuclei were stained with DAPI (Beyotime Biotechnology, Shanghai, China). Finally, images were acquired using a florescence microscope (Carl Zeiss, Oberkochen, Germany).

For immunofluorescence analysis, cells were washed with PBS and fixed in 4% paraformaldehyde for 10 min. The cells were then permeabilized with 0.5% Triton X-100 for 10 min, blocked in PBS containing 5% BSA for 30 min and stained with mouse anti-NP antibody for 1 h. After washing with PBS, the coverslips were incubated with the Alexa Fluor 488-labeled goat anti-mouse antibody (Beyotime). The nuclei were stained with DAPI. Finally, cells were washed with PBS and visualized under a florescence microscope.

### 2.6 Western blot assay

A549 cells were lysed in RIPA lysis buffer containing protease inhibitors (Beyotime). Equal amounts of proteins (20 μg/lane) were separated by 4–12% SDS-polyacrylamide gels (GenScript, Shanghai, China) and transferred onto nitrocellulose membranes using an eBlot L1 protein transfer system (GenScript). The membranes were blocked with 5% non-fat milk and incubated with primary antibodies at 4°C overnight. Then, the membranes were washed and incubated with horseradish peroxidase (HRP)-conjugated secondary antibodies for 1 h. The protein signals were detected with enhanced chemiluminescence detection kits (Thermo Scientific) and visualized by an Amersham Imager 600 (Cytiva Sweden AB, USA).

### 2.7 Coimmunoprecipitation and ubiquitination analysis

Coimmunoprecipitation and ubiquitination analyses were conducted in 293T cells. 293T cells transfected with the indicated plasmids and infected with the virus were lysed with RIPA buffer containing protease inhibitors. Ten percent of the lysates were kept as input controls, and the remaining lysates were incubated with specific antibodies. Antibody-protein complexes were further incubated with Protein A/G PLUS-Agarose (Santa Cruz Biotechnology) for 2–3 h. The agarose was thoroughly washed with lysis buffer and denatured with 5 × SDS-PAGE protein loading buffer at 96°C for 10 min. The samples and input were both subjected to Western blot analysis.

### 2.8 Statistical analysis

GraphPad Prism 9 software (San Diego, CA, USA) was used for the data analyses and graph creation. All the data are presented as the means ± standard deviations (SD) from at least three independent experiments. Statistical significance was calculated by a two-tailed *T*-test and a *p*-value of < 0.05 was considered significant.

## 3 Results

### 3.1 Identification of lncRNAs by SIV and SIV NS1 mutant virus infection

**FIGURE 1:**
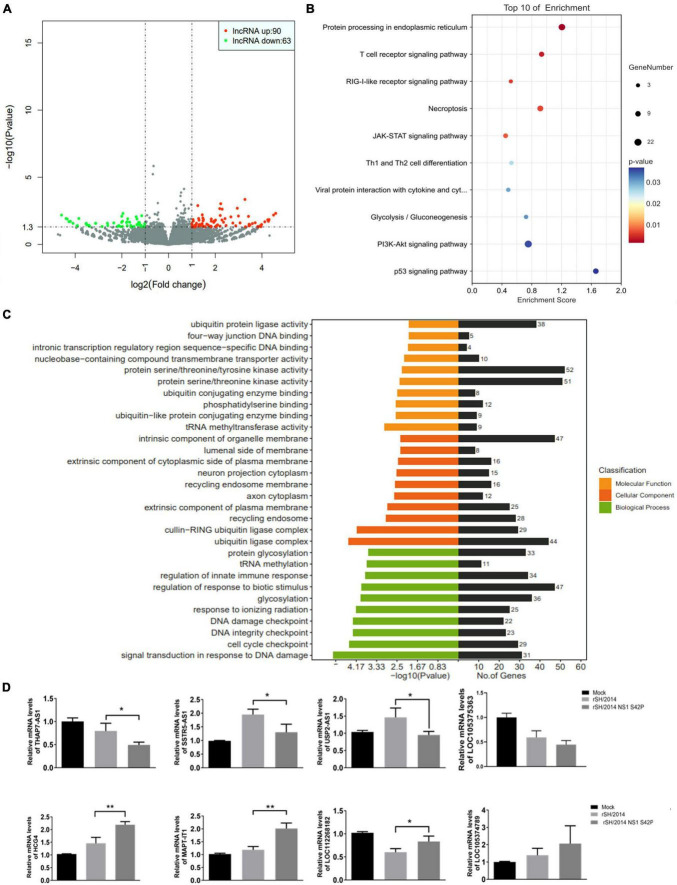
Host lncRNAs are differentially expressed during SIV and SIV NS1 mutant virus infection. **(A)** Volcano plot of differentially expressed lncRNAs in rSH/2014-infected or the mutant-infected A549 cells at an MOI of 1 for 24 h. Downregulated lncRNAs are denoted in green, and upregulated lncRNAs are denoted in red based on a *p*-value of ≤ 0.05 and a fold change of ≥2. **(B)** GO analysis includes 3 annotations: cellular components, molecular pathways and biological process of the differentially expressed lncRNAs. **(C)** Scatterplot of the top 10 most highly enriched KEGG pathways of predicted lncRNA targets. The *y*-axis represents pathway terms, and the *x*-axis (rich factor) represents the proportion of the targets accounting for all genes of a specific pathway term. The size of the point represents the gene count in this item. The *p*-values are color-coded. **(D)** The differential expression of 8 selected lncRNAs was confirmed by qPCR (*p < 0.05, **p < 0.01).

To explore the expression profile of lncRNAs in response to SIV infection, we performed high-throughput RNA-seq analysis on total cellular RNA obtained from A549 cells infected with rSH/2014 or rSH/2014 NS1 S42P, and compared the different lncRNA expression in these cells. According to the criteria (*p* < 0.05 and | log2FC| > 1), 90 upregulated lncRNAs and 63 downregulated lncRNAs were identified in NS1 mutant virus-infected cells compared with SH/2014-infected cells (Figure 1A and Supplementary File 1).

Based on Kyoto Encyclopedia of Genes and Genomes (KEGG) pathway analysis, these lncRNA targets were mapped to the T cell receptor signaling pathway, RIG-I-like receptor signaling pathway and JAK-STAT signaling pathway (Figure 1B). Gene Ontology (GO) analysis showed that these lncRNA targets were assigned to three categories of GO terms: “cellular component,” “biological process” and “molecular function” (Supplementary File 2). The most highly enriched biological process annotation involved “regulation of response to biotic stimulus,” “glycosylation” and “regulation of innate immune response” (Figure 1C). The bioinformatics results suggested that some of the expressed lncRNAs may regulate their neighboring genes, which are involved in the host immune response or IFN expression during SIV infection. Based on these data, 8 lncRNAs whose expression was significantly changed were selected for further validation by qRT-PCR, and the results were similar to the results obtained using RNA-seq (Figure 1D). Supplementary data to this article can be found online at https://www.ncbi.nlm.nih.gov/sra/PRJNA1011353.

### 3.2 HCG4 upregulation during SIV NS1 mutant infection over time

**FIGURE 2:**
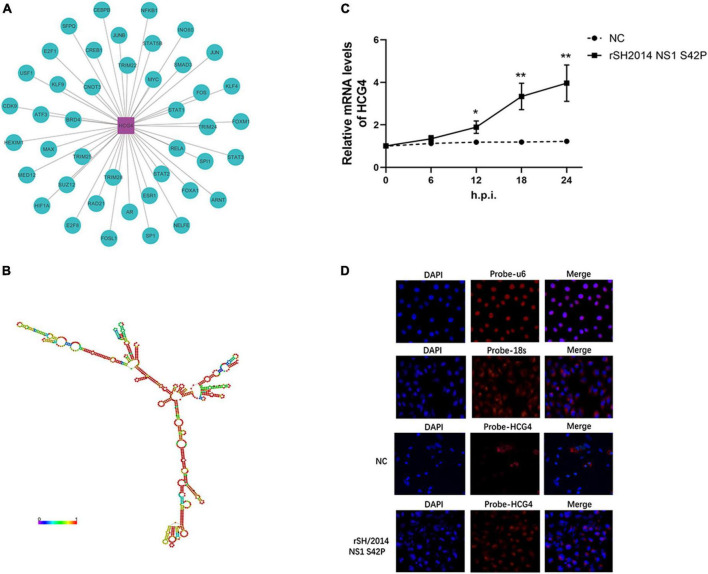
Further characterization of HCG4. **(A)** The potential transcriptional factors (TFs) of HCG4 were analyzed via the TRANSFAC database. **(B)** RNA secondary structure prediction for lncRNA HCG4 was analyzed using RNAfold web server. **(C)** A549 cells were infected with rSH2014 NS1 S42P, and harvested at indicated time points after infection. The expression levels of HCG4 were tested by qPCR (*p < 0.05, **p < 0.01). **(D)** FISH analysis of HCG4 (red) in rSH2014 NS1 S42P-infected A549 cells at 24 h.p.i. compared with uninfected group (NC). The nuclei were stained with DAPI. U6 and 18S probes hybridized in A549 cells were used as nuclear and cytoplasmic markers, respectively.

The RNA-seq results showed that MAPT-IT and HCG4 were the most significantly changed host lncRNAs at 24 h.p.i. with SIV NS1 mutant infection, as they were showed higher level in the rSH/2014 NS1 S42P infection group than that in the rSH/2014 infection group (*p* < 0.01). Moreover, when using TRANSFAC to analyze the associated transcription factors of HCG4, we found that STAT1/2/3 was highly related with HCG4 (Figure 2A). Therefore, the lncRNA HCG4 was selected for in-depth study. HCG4 is located in chromosome 6p22.1 of the human genome. According to the RNAfold webserver, HCG4 was predicted as a highly folded secondary structure containing several hairpin loops (Figure 2B). Then, we determined the trends in HCG4 expression changes during NS1 mutant virus infection over time by qRT-PCR, and the results showed marked upregulation from 12 h.p.i. onward (Figure 2C). FISH with probes specific for HCG4 was performed, and the results confirmed increased HCG4 expression in the cytoplasm at 24 h.p.i. upon NS1 mutant virus infection (Figure 2D).

### 3.3 HCG4 overexpression inhibited SIV infection

**FIGURE 3:**
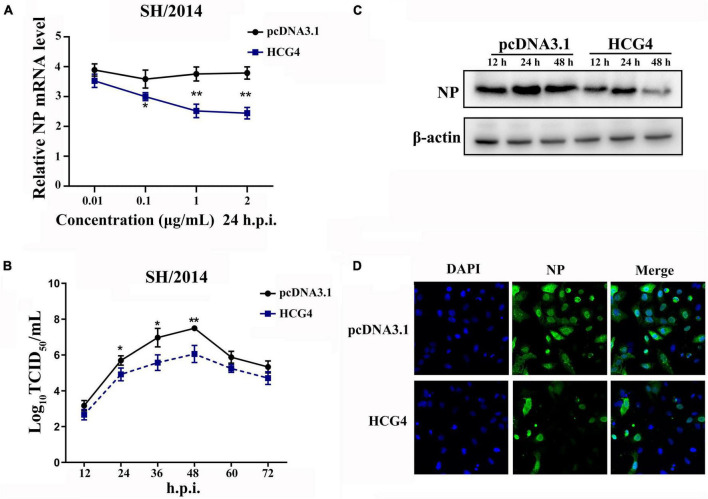
Overexpression of HCG4 inhibited SIV infection. **(A)** A549 cells were transfected with indicated concentrations of the plasmid encoding HCG4 or control vector and then infected with SH/2014 at an MOI of 1. SIV NP mRNA levels were detected by qPCR at 24 h.p.i. A549 cells were transfected with 1 μg/ml HCG4 or control vector and then infected with SH/2014 at an MOI of 1. The cellular supernatants were collected at the indicated time points post-infection, and the viral load was quantified based on TCID_50_ on MDCK cells **(B)** NP protein expression levels were tested by Western-blotting **(C)** and indirect immunofluorescence **(D)**. (*p < 0.05, **p < 0.01).

To investigate the effect of HCG4 on viral replication, A549 cells were transfected with plasmids encoding HCG4 and subsequently infected with SH/2014 to test the effect of HCG4. As shown in Figure 3A, HCG4 overexpression significantly inhibited the mRNA level of viral NP at 24h.p.i. Moreover, it was suppressed by HCG4 in a dose-dependent manner, manifesting as the gradually reduced NP mRNA level along with the increased amount of HCG4 transfected plasmid. The TCID_50_ value showed that overexpression of HCG4 inhibited viral replication of SH/2014. Specifically, the viral titer of SH/2014 was decreased 25.11-fold at 36 h.p.i and 27.54-fold at 48 h.p.i. (Figure 3B). Western blotting and indirect immunofluorescence analysis of NP protein further confirmed an inhibitory effect on SIV replication (Figures 3C, D).

### 3.4 HCG4 knockdown promoted SIV infection

**FIGURE 4:**
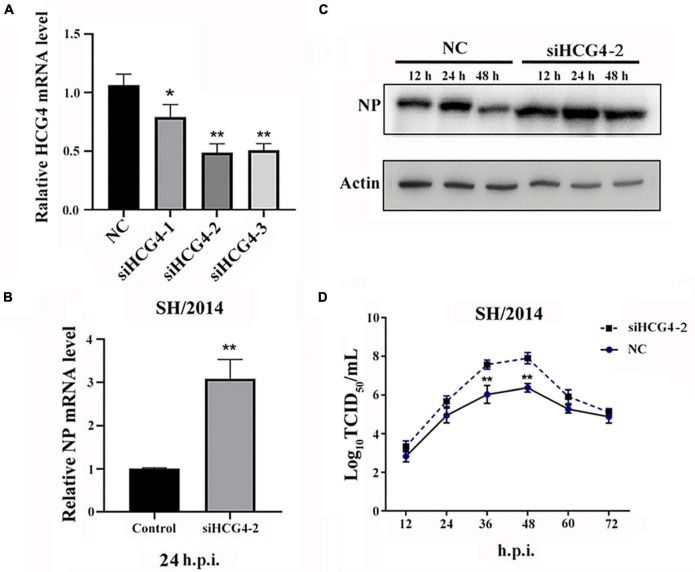
Knockdown of HCG4 promoted SIV infection. **(A)** A549 cells were transfected with 100 nM of different siRNAs targeting HCG4 or NC siRNA, and their interference efficiency was measured by qRT-PCR. **(B)** A549 cells were transfected with 100 nM siHCG4-2 or NC siRNA and subsequently infected with SH/2014 at an MOI of 1. SIV NP protein mRNA levels were tested by qPCR at 48 h.p.i. **(C)** Cell lysates were collected at the indicated time points post-infection for Western blot analysis of NP protein expression. **(D)** Cellular supernatants were collected at the indicated time points post-infection, and the viral load was quantified based on the TCID_50_ in MDCK cells. (*p < 0.05, **p < 0.01).

For the loss-of-function assay, small interfering RNAs (siRNAs) specifically targeting HCG4 were used to knock down endogenous HCG4 expression in A549 cells. We designed 3 siRNAs, and the expression level of HCG4 was effectively reduced by two of the siRNAs, as assessed by qRT-PCR after RNA interference (*p* < 0.01). As a control, cells were transduced in parallel with a non-targeting siRNA (Figure 4A). In concert with the previous results in the overexpression assay, we found that in A549 cells transfected with siHCG4, NP mRNA levels were increased by approximately threefold compared to those in the control cells at 24 h.p.i. (Figure 4B). Similarly, elevated expression of NP was observed in HCG4 knockdown cell (Figure 4C). Moreover, the viral titer of SH/2014 was increased at 36 and 48 h.p.i. (Figure 4D). These results showed that endogenous HCG4 downregulation promotes SIV replication, further supporting the antiviral role of HCG4 in the host.

### 3.5 HCG4 reduce the ability of SIV to antagonize IFN through RIG/IRF3 signaling

**FIGURE 5:**
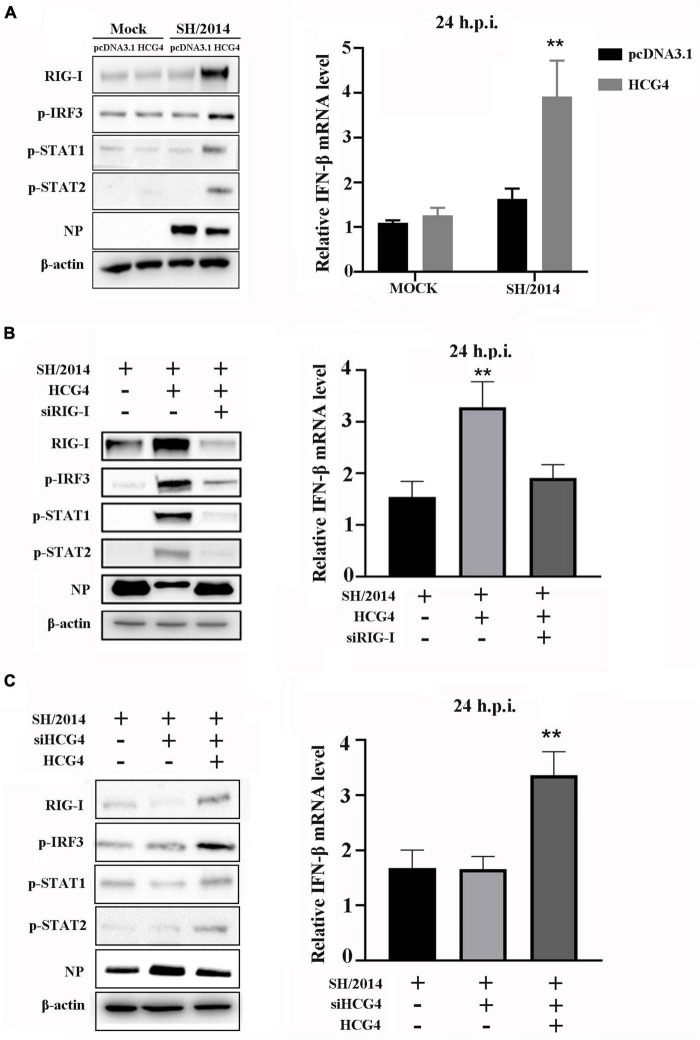
RIG-I mediates HCG4-induced IFN expression. **(A)** A549 cells were transfected with control vector or plasmid encoding HCG4 (1 μg/ml). After 24 h, cells were left uninfected infected with SH/2014 at an MOI of 1 for 24 h. **(B)** A549 cells were transfected with control vector or plasmid encoding HCG4 or co-transfected with plasmid encoding HCG4 and siRNA targeting RIG-I. After 24 h, cells were infected with SH/2014 for 24 h. **(C)** A549 cells were transfected with siRNA targeting HCG4. After 48 h, cells were transfected with control vector or plasmid encoding HCG4. Then, cells were infected with SH/2014 for 24 h. The protein level of RIG-I, p-IRF3, p-STAT1, p-STAT2, viral NP and β-actin were analyzed using Western blotting. The relative mRNA level of IFN-β was analyzed using qPCR. The error bars represent the means plus standard deviations for three independent experiments. (**p < 0.01).

In a previous study, we demonstrated that the mutant virus rSH/2014 NS1 S42P could activate RIG/IRF3 pathway, which led to high levels of IFN production (Cheng et al., 2018). To determine whether HCG4 regulates RIG/IRF3 activation and thus induces IFN production, A549 cells were transfected with the HCG4 and then infected with SIV. As shown in Figure 5A, in HCG4 overexpressed cells, SH/2014 infection increased the expression of RIG-I, p-IRF3, and p-STAT1/2, and the IFN-β level was also significantly increased compared to that in the control cells. Next, we assessed the effect of the loss of RIG-I on IFN production in HCG4 overexpressed cells. When RIG-I was knocked down, HCG4 did not cause increases in p-IRF3 and p-STAT1/2 levels or did it cause an increase in IFN-β production.

To further elucidate the contribution of HCG4 to RIG/IRF3 pathway signaling, we assessed RIG-I, p-IRF3, p-STAT1/2, and IFN-β levels in the presence of HCG4 siRNA transfection. The results showed that HCG4 knockdown restricted the expression of RIG-I, p-IRF3, and p-STAT1/2, as well as IFN-β production, compared to the control group (Figure 5B). However, when the HCG4 knockdown cells were transfected with plasmids encoding HCG4, the protein levels of RIG-I, p-IRF3, and p-STAT1/2 and IFN-β production were restored (Figure 5C).

### 3.6 HCG4 impairs NS1 suppression of RIG-I ubiquitination

**FIGURE 6:**
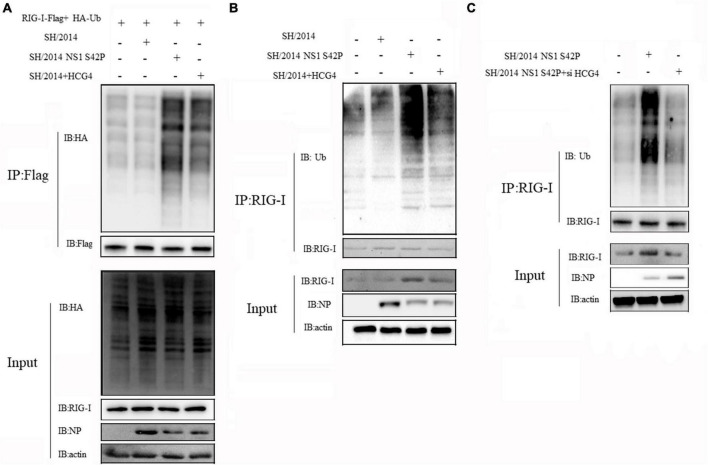
HCG4 promoted K63-linked RIG-I polyubiquitination. **(A)** 293T cells were co-transfected with Myc-RIG-I, HA-Ub and HCG4 for 24 h and followed by infection with rSH/2014 or rSH/2014 NS1 S42P at an MOI of 1 for 24 h. Cell lysates were precipitated with anti-Myc antibodies and Western-blot analyzed the K63-linked ubiquitination of RIG-I. **(B)** 293T cells were transfected with HA-Ub for 24 h, and then infected with rSH/2014 or rSH/2014 NS1 S42P at an MOI of 1 for 24 h. Cell lysates were precipitated with anti-RIG-I antibody, followed by Western-blot with anti-HA antibody to analyze the K63-linked ubiquitination of RIG-I. **(C)** 293T cells were interfered with siRNA specific for HCG4 or scrambled siRNA for 24 h followed by HA-Ub transfection for 24 h, then infected with rSH/2014 NS1 S42P at an MOI of 1 for 24 h. Cell lysates were precipitated with anti-RIG antibody, and Western blotting was used to analyze the ubiquitination of RIG-I.

Given that influenza A virus NS1 specifically inhibits RIG-I CARD ubiquitination, resulting in the suppression of antiviral signal transduction, we postulated that HCG4 might specifically alleviate this step to promote RIG-I activation. To this end, immunoprecipitation and Western blotting were conducted to examine the effect of HCG4 expression on RIG-I ubiquitination. We cotransfected 293T cells with plasmids expressing Myc-RIG-I and HA-Ub and compared the capacity of rSH/2014 and NS1 mutant virus to promote RIG-I K63-linked ubiquitination. As shown in Figure 6A, rSH/2014 NS1 S42P infection promoted RIG-I K63-linked ubiquitination compared to rSH/2014, and then when cells were transfected with HCG4, the ubiquitination levels of RIG-I began to rise in cells infected with rSH/2014. Furthermore, an endogenous experiment was conducted in which the cell lysates were immunoprecipitated with anti-RIG-I antibodies. As predicted, upon rSH/2014 infection, cells transfected with the HCG4 exhibited an increase in endogenous RIG-I K63-linked ubiquitination compared to cells transfected with the empty vector (Figure 6B). Subsequently, we investigated the K63-linked ubiquitination of RIG-I upon NS1 mutant virus infection in the absence of HCG4. The results showed that the K63-linked ubiquitination level of RIG-I in cells was at a high level similar to infection with NS1 mutant virus rSH/2014 NS1 S42P, but it decreased in HCG4 knockdown cells (Figure 6C). Collectively, these results suggest that HCG4 regulates K63-linked RIG-I ubiquitination, potentially activating the RIG-I pathway and downstream signal transduction.

## 4 Discussion

Influenza A virus NS1 is a highly expressed protein that has multiple regulatory functions during virus infection. The presence of serine at position 42 (S42) in NS1 has been shown to be essential in affecting antiviral cytokine responses in many different subtypes of the influenza virus (Donelan et al., 2003; Jiao et al., 2008; Hsiang et al., 2012). Previously, we reported that S-to-P substitution of NS1 impairs IFN inhibition by blocking the activation of IRF3 and thus facilitates the virus. Here, by lncRNA-seq, we found that the cellular levels of many lncRNAs were differentially expressed when the amino acid was mutated at position 42 of the SIV NS1 protein. We further explored lncRNAs that are characterized as IFN antagonists of the NS1 protein and unveiled their role in host-pathogen interactions during IAV infection.

As lncRNAs emerge as important regulators of cellular processes, many differentially expressed lncRNAs have been reported after IAV infection, and their potential roles in pathogenesis and their functions in host cells have been progressively unveiled. For example, some lncRNAs have been demonstrated to affect IAV RNP complex activity to promote or inhibit viral replication. Wang et al. reported that the host lncRNA IPAN is hijacked by IAV to assist virus replication by stabilizing the viral RNA polymerase PB1 (Wang et al., 2019). A study by Zhang et al. (2021) showed that lnc45 could be induced by various subtypes of IAV, suppressing the polymerase activity and nuclear accumulation of PA and NP, which attenuated virulence. On the other hand, lncRNAs can also regulate the expression of some genes or proteins in host cells to affect IAV propagation. lncRNA NRAV was shown to negatively regulate IFITM3 and MxA expression by affecting histone modification of these genes, which play a critical role in anti-IAV infection (Ouyang et al., 2014). In addition, some lncRNAs have been shown to target host metabolic pathways for virus replication or escape from host defenses. Wang et al. (2017) reported that virus-induced lncRNA ACOD1 enhanced the catalytic activity of the metabolic enzyme glutamic-oxaloacetic transaminase (GOT2), which partly altered the metabolic network of the host, exerting broad-spectrum antiviral activity. In this study, we demonstrate the antiviral role of the lncRNA HCG4, one of the most highly induced lncRNAs after NS1 mutant infection. Functional analysis showed that HCG4 overexpression caused high level of IFN production in response to SIV infection, and the amount of progeny virus in the supernatant was reduced, indicating the inhibitory effect of HCG4 on SIV replication.

Virus-triggered IFNs induction plays a crucial role in the antiviral response by activating the JAK-STAT pathway. Once IFNs binds to cell surface receptors, the signal is transmitted through the membrane and into the cell, and subsequently signal transducer and activator of transcription (STAT) proteins are recruited to the receptor, phosphorylated then result in numerous IFN stimulated genes (ISGs) to inhibit viral replication and establish an antiviral state (Schneider et al., 2014; Hoffmann et al., 2015). Because of its significance in immune response, viruses have developed strategies target STAT proteins to evade the defense during the infection, including porcine reproductive and respiratory syndrome virus (PRRSV), HCV, Ebola Virus that suppresses IFN signaling by leading to phosphorylated STAT1 reduction (Lin et al., 2006; Reid et al., 2006; Yang and Zhang, 2017). Consistent with the role of IAV NS1 protein exerts its inhibitory effects on IFNs production, phosphorylation of STATs proteins was strongly reduced in cells infected with IAV (Pauli et al., 2008; Jia et al., 2010). In our study, KEGG pathway analysis showed that JAK-STAT pathway was highly enriched by NS1 mutant virus infection. Meanwhile, we demonstrated SIV infection inhibits IFN-β production and STAT1/2 phosphorylation, however, after overexpression with HCG4, the levels of IFN-β and phosphorylated STAT1 and STAT2 increased significantly. Therefore, we conclude that HCG4 relieved the inhibition of IFN-β production as well as endogenous STAT1/2 phosphorylation. Moreover, when RIG-I was interfered in cells, forced HCG4 expression could not cause the increase in IFN-β production, nor the up-regulation of stat1/2 phosphorylation. We supposed that the HCG4 served as a positive feedback regulator through RIG-I mediated IFN signaling during SIV infection, thus exerts anti-viral effects to SIV.

Ubiquitination is a prominent type of post-translational modification that regulate the signal transduction pathways of innate antiviral immune responses (Zinngrebe et al., 2014). RLR signal transduction is also regulated by ubiquitination upon RNA virus infection and different types of ubiquitination by E3 ubiquitin ligases have different outcomes (Gu and Jan Fada, 2020). For example, K48-linked ubiquitination of the adaptor proteins guides to proteasome-dependent degradation whereas K63-linked ubiquitination promotes the activation of these molecules for downstream signaling. As a cellular sensor, the K63-linked ubiquitination of RIG-I is indispensable to initiate signaling cascade and finally induces IFN production. However, many viruses, have evolved strategies by targeting RIG-I to evade the innate immune response. For example, PRRS N protein, Human respiratory syncytial virus (HRSV) NS1 protein, and the V proteins of several members of the family Paramyxoviridae can suppress RIG-I ubiquitination and subsequent RIG-I mediated antiviral signaling (Ban et al., 2018; Sánchez-Aparicio et al., 2018; Zhao et al., 2019). In particular, the IAV NS1 protein is the main IFN antagonist and was shown to specifically inhibits RIG-I ubiquitination. Some truncated or mutated amino acids in the NS1 proteins results in a deficit in NS1’s ability to antagonize RIG-I ubiquitination. For example, NS1 R21Q mutation or a deletion of EALQR motif at 191 to 195 could diminish inhibition of RIG-I ubiquitination (Wang et al., 2018; Jureka et al., 2020). In this study, we observed that SIV NS1 S-to-P mutation is less able to inhibit RIG-I K63-linked ubiquitination. Meanwhile, when cells transfected with the HCG4 gene, there is an increase in RIG-I ubiquitination upon SIV infection. Furthermore, the level of RIG-I ubiquitination decreased upon NS1 S42P mutant virus infection in HCG4 knockdown cells. These results suggest that HCG4 positively regulates K63-linked RIG-I ubiquitination, potentially facilitates the downstream signal transduction and the IFN induction.

In conclusion, our findings suggested that lncRNA HCG4 significantly elevated after SIV NS1 mutant infection, and this is the first study to describe the role of lncRNA HCG4 for NS1 function in SIV infection. This work reveals lncRNA HCG4 as a positive regulator in RIG-I mediated antiviral interferon response, which provides new insights into roles of lncRNAs in virus-host interactions.

## Data availability statement

The datasets presented in this study can be found in online repositories. The names of the repository/repositories and accession number(s) can be found in the article/Supplementary material.

## Ethics statement

Ethical approval was not required for the studies on humans in accordance with the local legislation and institutional requirements because only commercially available established cell lines were used. Ethical approval was not required for the studies on animals in accordance with the local legislation and institutional requirements because only commercially available established cell lines were used.

## Author contributions

JC: Data curation, Funding acquisition, Writing – original draft, Writing – review and editing. JT: Methodology, Resources, Writing – review and editing. BL: Formal analysis, Writing – review and editing. YS: Methodology, Writing – review and editing. HL: Conceptualization, Supervision, Writing – review and editing.
